# Membrane Rearrangements in the Maturation of Circulating Human Reticulocytes

**DOI:** 10.3389/fphys.2020.00215

**Published:** 2020-03-17

**Authors:** Giampaolo Minetti, Claudia Bernecker, Isabel Dorn, Cesare Achilli, Stefano Bernuzzi, Cesare Perotti, Annarita Ciana

**Affiliations:** ^1^Laboratories of Biochemistry, Department of Biology and Biotechnology “L. Spallanzani”, University of Pavia, Pavia, Italy; ^2^Department of Blood Group Serology and Transfusion Medicine, Medical University of Graz, Graz, Austria; ^3^Servizio Immunoematologia e Medicina Trasfusionale, Fondazione Istituto di Ricovero e Cura a Carattere Scientifico Policlinico San Matteo, Pavia, Italy

**Keywords:** membrane rafts, membrane skeleton, Band 3, flotillin, stomatin, cultured red blood cells, lipidomics, Western blotting

## Abstract

Red blood cells (RBCs) begin their circulatory life as reticulocytes (Retics) after their egress from the bone marrow where, as R1 Retics, they undergo significant rearrangements in their membrane and intracellular components, via autophagic, proteolytic, and vesicle-based mechanisms. Circulating, R2 Retics must complete this maturational process, which involves additional loss of significant amounts of membrane and selected membrane proteins. Little is known about the mechanism(s) at the basis of this terminal differentiation in the circulation, which culminates with the production of a stable biconcave discocyte. The membrane of R1 Retics undergoes a selective remodeling through the release of exosomes that are enriched in transferrin receptor and membrane raft proteins and lipids, but are devoid of Band 3, glycophorin A, and membrane skeletal proteins. We wondered whether a similar selective remodeling occurred also in the maturation of R2 Retics. Peripheral blood R2 Retics, isolated by an immunomagnetic method, were compared with mature circulating RBCs from the same donor and their membrane protein and lipid content was analyzed. Results show that both Band 3 and spectrin decrease from R2 Retics to RBCs on a “per cell” basis. Looking at membrane proteins that are considered as markers of membrane rafts, flotillin-2 appears to decrease in a disproportionate manner with respect to Band 3. Stomatin also decreases but in a more proportionate manner with respect to Band 3, hinting at a heterogeneous nature of membrane rafts. High resolution lipidomics analysis, on the contrary, revealed that those lipids that are typically representative of the membrane raft phase, sphingomyelin and cholesterol, are enriched in mature RBCs with respct to Retics, relative to total cell lipids, strongly arguing in favor of the selective retention of at least certain subclasses of membrane rafts in RBCs as they mature from Retics. Our hypothesis that rafts serve as additional anchoring sites for the lipid bilayer to the underlying membrane-skeleton is corroborated by the present results. It is becoming ever more clear that a proper lipid composition of the reticulocyte is necessary for the production of a normal mature RBC.

## Introduction

Reticulocytes (Retics) are the result of the enucleation in the bone marrow of their immediate nucleated precursor, the orthochromatic erythroblast, and still retain several components in their membrane and cytoplasm with an excess of approximately 20% of plasma membrane that must be removed. Part of the maturation occurs in the bone marrow, where the cells are defined as R1 Retics, part in the circulation (R2 Retics) where they will lose additional components and residual excess membrane before they attain the final structure of a fully functional and stable biconcave discocytic red blood cell (RBC) (Chasis et al., 1989; Moras et al., 2017; Ovchynnikova et al., 2018). In turn, RBCs lose membrane surface area and cell volume as they age in the circulation. It is common belief that surface area is lost through the release of vesicles. Vast literature is available on the characterization of vesicles released *in vitro* under a variety of treatments: nutrient deprivation, increased intracellular Ca^2+^, pH changes, intercalation of amphiphilic compounds in the membrane, etc. The vast majority of types of *in vitro*-released vesicles are defined as being spectrin-free (devoid of membrane skeleton). If the vesicles that are supposedly released *in vivo* were also spectrin-free, young and old RBCs should contain the same amount of spectrin. We have recently shown that this is not the case, as the membrane skeleton is apparently lost in parallel with the lipid bilayer during RBC aging (Ciana et al., 2017a, b), suggesting that the membrane is lost by/removed from aging RBCs in a form that is different from the well-known and characterized spectrin-free vesicles obtained *in vitro*. A recent model proposes that, in the peculiar environment of the oscillatory splenic flow, conditions may arise whereby RBCs can spontaneously release vesicles through a novel deformation mode, called “infolding” (Asaro et al., 2018). Support to this theoretical model from experimental evidence obtained *in vivo* is still lacking. Also unknown is the mechanism by which the membrane and the membrane skeleton are removed, but probably require the active intervention of other tissues/organs (spleen, liver, endothelium).

When looking at the membrane of young and old circulating RBCs, we have found that flotillin-2, a membrane raft component, is lost disproportionately with respect to the loss of membrane that takes place during RBC aging (more flotillin is lost than surface area extension). This suggests that selected portions of the membrane, enriched in flotillins are shed/removed during the physiological aging of the cell. Interestingly, when looking at the partitioning of flotillin-2 between the membrane of the cell and that of the vesicles released *in vitro* by Ca^2+^-treatment, the protein is found to be depleted in the vesicles, again pointing to a different mechanism of membrane shedding *in vivo* and *in vitro* (Ciana et al., 2017a, b). This evidence suggested that membrane rafts are involved in the selective processing of the plasma membrane as RBCs age *in vivo*.

Membrane rafts are already known to be involved in the maturation of R1 Retics in the bone marrow after enucleation of the orthochromatic erythroblast. Recent advances in the field of erythropoiesis and the production of cultured RBCs, have clearly shown that an important role is played by membrane lipids, in particular cholesterol, in the differentiation and maturation process (Bernecker et al., 2019; Gu et al., 2019; Zingariello et al., 2019). With the discovery of the multivesicular body (MVB), it became clear that certain proteins are retained in the membrane (Band 3, glycophorin A, all membrane-skeletal proteins), while others (transferrin receptor, TfR or CD71) are lost in exosomes that result from membrane trafficking that starts at the plasma membrane, leads to the endosomal compartment, then to the multivesicular body [(containing intraluminal-vesicles (ILV)] and finally to the extracellular space (Pan and Johnstone, 1983; Harding et al., 1984). Only in more recent years, and after the notion of membrane rafts became popular, it was observed that exosomes are also enriched in membrane raft components (de Gassart et al., 2003). Despite the much advancement in this field, it could be said that the study of Retic maturation is still in its infancy. In fact, many questions concerning the major pathways of erythroblast maturation and their overlapping, especially around the terminal maturation of the Retic, are still unanswered (Ney, 2011). For instance, it is still unclear: (i) why the TfR, that is endocytosed in clathrin-coated vesicles (that do not contain membrane rafts) is then found in the exosomes together with membrane raft components; (ii) to what extent cultured Retics can mature *in vitro*: although the erythroblasts spontaneously enucleate *in vitro*, do the resulting Retics also traffic membrane via the MVB and release exosomes?; (iii) how R2 Retics lose an extra amount of membrane and the residual TfR to become mature discocytes; (iv) how selective is the loss of membrane components from R2 Retics?; (v) is there any loss of membrane-skeleton from R2 Retics? and (vi) how the lipid composition of the membrane bilayer changes all along erythroid differentiation and in particular in the maturation of Retics (both R1 and R2) (Minetti et al., 2018).

We reasoned that, if membrane rafts are important in the two above-described steps (aging of mature RBCs in the circulation and maturation of R1 Retics in the marrow), they could also be involved in the terminal maturation of circulating (R2) Retics. We therefore approached the characterization of the membrane of circulating Retics and RBCs from the same donor. Thus, we focused on flotillins and stomatin as selected protein markers of rafts, aiming at evaluating whether a selective sorting of these raft components in Retics and RBCs could be detected. Membrane rafts are defined as being enriched in cholesterol and sphingolipids (Sonnino et al., 2006). If a rearrangement of the membrane rafts phase occurred in the terminal maturation of Retics *in vivo*, this should be reflected by changes in the lipid composition of the plasma membrane. Because of this, and in the light of the ever increasing importance that lipids appear to have in erythropoiesis (Bernecker et al., 2019; Zingariello et al., 2019), lipidomics analysis of R2 Retics and mature RBCs was carried out. Answering these basic questions could help expand our knowledge of erythropoiesis and the mechanism(s) of clearance of normal and pathological RBCs, and to find new solutions for the production of fully mature and functional cultured RBCs (Anstee et al., 2012).

## Materials and Methods

Human blood samples were collected in standard 6 ml vials containing lithium heparin as the anticoagulant by the local Transfusion center (Servizio di Immunoematologia e Medicina Trasfusionale of the IRCCS Policlinico San Matteo, Pavia, Italy) from regular donors after informed consent was obtained, according to the protocol approved on 2017/04/10, by the local ethics committee (Comitato Etico Area Pavia, IRCCS Policlinico San Matteo, Pavia, Italy). Processing of blood and blood cells was terminated within the day of withdrawal.

### Blood Filtration, RBC, and Retic Recovery

The blood was filtered to separate RBCs from white blood cells and platelets (Beutler et al., 1976; Achilli et al., 2011). Whole blood (8–10 ml) was centrifuged at 1000 × *g* for 5 min to sediment the cells. One milliliter of plasma was collected and diluted with 9 ml of PBSG [5 mM sodium phosphate pH 7.4, 154 mM NaCl, 4.5 mM KCl, 305–310 mosmol/kg H_2_O (osmolality was measured with a freezing point depression osmometer Micro-Osmometer Type 13/13 DR Roebling, Berlin, Germany) supplemented with 10 mM glucose before use]. The remaining plasma was collected and set aside. The diluted plasma was used to condition the cellulose filter through which blood was filtered. The filter was contained in a plastic Buchner funnel (*d* = 5.2 cm internal) and was formed by a cellulose bed. Six gram of a mixture of equal parts, by weight, of α-cellulose (Merck Sigma-Aldrich code C8002) and microcrystalline cellulose (Merck Sigma-Aldrich code S5504) were suspended in about 60 ml of PBSG and poured into the Buchner funnel whose bottom was lined with a disc of Whatman grade 4 filter paper (that holds particles larger than 20–25 μm). The cellulose bed was washed with 100 ml of PBSG to ensure the washing out of finer cellulose particles (<20–25 μm) that would co-elute with, and contaminate RBCs during filtration. The packed RBCs left in the tube after removal of the plasma were resuspended to approximately 50% hematocrit (Ht) with PBSG and the suspension was uniformly distributed on the cellulose filter. Once the RBCs entered the cellulose, PBSG was added and the eluate, containing the filtered RBCs was collected in 50 ml polypropylene centrifuge tubes until no more RBCs emerged from the filter. Through two washes with PBSG and centrifugation for 5 min at 1000 × g at 20°C, the contents of all the tubes was combined into a single tube, which was again centrifuged. Sufficient PBSG was removed to bring the Ht to approximately 20% and the volume of the resulting suspension and its Ht were measured. Some experiments were dedicated to evaluate the yield of Retics after filtration. Aliquots of whole blood and of filtered RBCs as suspensions of approximately 20% Ht, were counted for Retic number with an Advia 2120 Automated Hematology analyzer (Siemens, Munich, Germany) at the “Laboratory of Analysis,” IRCCS Foundation, Policlinico San Matteo, Pavia.

### Immuno-Magnetic Separation of Retics

This method was used for the positive selection and purification of Retics using “CD71 MicroBeads” (conjugated to monoclonal anti-human CD71 antibodies, isotype: mouse IgG2a; clone AC108.1, MACS Miltenyi Biotec, United States). The procedure was carried out at 4°C. Filtered RBCs (3.2 ml packed cells) were brought to 20% Ht with PBSG containing 0.5% (w/v) BSA for a final volume of 16 ml. To this suspension, 600 μl of “CD71 MicroBeads” were added and the mixture was rotated end-over-end for 45 min. Extension of incubation time from the 15 min suggested by the manufacturer to 45 min proved to be essential to increase Retic recovery. One magnetic column of the type MS (Medium size, Miltenyi Biotec, code 130-042-201) or, in later experiments LS (Large size, Miltenyi Biotec, code 130-042-401) was inserted into the permanent magnet of a MiniMACS^TM^ or MidiMACS^TM^ separator (Miltenyi Biotec). Four such columns were used per experiment, with blood from a single donor, expecting to recover from 10^7^ to 10^8^ Retics in total from each experiment. Three milliliter of PBSG + BSA were passed through each column to condition it. Then, 4 ml of RBC suspension + “CD71 Microbeads” were loaded on each column and the eluate was collected and saved. Three milliliter PBSG + BSA were then added to wash out the remaining RBCs. Retic recovery was also increased by reducing the flow rate during both loading of the cell suspension on the column and washing of the column before eluting the Retics. Reduction of flow rate was obtained by attaching a small-gauge needle to the Luer-slip fitting of the column. The column was then extracted from the magnet and 1.5 ml of PBSG were added to elute, with the aid of a plunger, the Retics in a 2 ml Eppendorf tube. Retics were sedimented for 4 min at 1000 × g. The supernatant was discarded and the Retics were resuspended with PBSG (final volume approximately 55–60 μl). Five microliter of this suspension were set aside for Retic staining and counting. The remaining suspension was destined for Western blotting or lipid analysis and processed as follows.

### Sample Preparation for Western Blotting

Fifty microliter of Retic suspension were mixed with 450 μl of “Diluted SDS-PAGE sample Buffer” [prepared by mixing 1 volume of 3X SDS-PAGE sample buffer (50 mM Tris/HCl, pH 6.8, 5% SDS (w/v), 35% sucrose (w/v), 5 mM EDTA, 0.01% bromophenol blue, 200 mM dithiotreitol) with 1.7 volumes of 5% SDS (w/v) in MilliQ water] and the samples were incubated at 60°C for 15 min. Samples were stored frozen at −80°C in 100 μl aliquots until analysis by SDS-PAGE and Western Blotting.

### Sample Preparation for Lipid Analysis

Five, out of a 50 μl of Retic suspension were mixed with 45 μl of “Diluted SDS-PAGE sample Buffer” (see above), treated at 60°C for 15 min and stored frozen for Western blotting analysis. To the remaining 45 μl of Retic suspension, 1 ml of ice-cold, HPLC-grade methanol was added, the sample transferred into Pyrex^®^ glass tubes (Code: 1636726MP, capacity 11 ml, SciLabware Ltd., United Kingdom) and stored frozen at −80°C until analysis.

### Cell Count

In some experiments Retics and RBCs were manually counted in Neubauer chamber after suitable dilution of the stock cell suspensions with PBSG + BSA.

### Lipid Analysis

The quantitative lipid measurement was done by high resolution mass spectrometry (LTQ-Orbitrap, Thermo Scientific) (Triebl et al., 2017). Lipids were extracted from cell pellets (10^7^–10^8^ cells) with an established MTBE (Methyl-tert-butylether) protocol (Matyash et al., 2008). The Orbitrap Velos Pro hybrid mass spectrometer was operated in Data Dependent Acquisition mode using a HESI II ion source. Full scan profile spectra from m/z 450 to 1050 for positive ion mode and from m/z 400 to 1000 for negative ion mode were acquired in the Orbitrap mass analyzer at a resolution of 100k at m/z 400 and <2 ppm mass accuracy. Samples were measured once in positive polarity and once in negative polarity. For MS/MS experiments, the ten most abundant ions of the full scan spectrum were sequentially fragmented in the ion trap using He as collision gas (CID, Normalized Collision Energy: 50; Isolation Width: 1.5; Activation Q: 0.2; and Activation Time: 10). Centroided product spectra at a normal scan rate (33 kDa/s) were collected. The custom developed software tool Lipid Data Analyzer was used for data analysis (Hartler et al., 2011; Hartler et al., 2017). Seven classes of lipids were quantified, together with their subclasses according to the length and number of double bonds of the acyl chains linked to the glycerol or sphingosine moiety: phosphatidylcholine (PC), lysophosphatidylcholine (LPC), sphingomyeline (SM), phosphatidylethanolamine (PE), phosphatidylserine (PS), phosphatidylinositol (PI), and cholesterol (Chol).

### SDS-PAGE and Western Blotting

RBC and Retic proteins were separated by Polyacrylamide Gel Electrophoresis in Sodium DodecylSulfate (SDS-PAGE), following the Laemmli method (Laemmli, 1970), in 10% acrylamide isocratic (or, for analysis of spectrin, 5–15% acrylamide linear gradients) mini-gels (80 × 60 × 1.5 mm) in a Mini Protean 3 system (Bio-Rad Laboratories, United States). Sample loading is indicated in the Results section or in figure legends.

Proteins separated by the SDS-PAGE were electro-transferred to a PVDF membrane (0.2 μm pores) using a Trans Blot Turbo system (Bio-Rad Laboratories, United States) according to manufacturer’s instructions. Membranes were blocked with a blocking solution [5% skimmed milk in 20 mM Tris, pH 7.4, 150 mM NaCl, 0.05% Tween 20 (v/v)] and then incubated overnight at 4°C with the primary antibody directed to the protein of interest (Table 1).

**TABLE 1:** Primary and secondary antibodies used (at the dilution indicated in parentheses) for Western blotting in the present study.

**Primary antibodies**	**Secondary antibodies**
Anti human flotillin-2, mouse polyclonal, Abnova H00002319-B02P (1:3000).	HRP-conjugated goat anti-mouse IgGs, ENZO Life Sciences BML-SA204 (1:5000)
Anti human stomatin, goat polyclonal (M-14), SCBT sc-48308 (1:3000)	HRP-conjugated donkey anti-goat IgGs, SCBT sc-2020 (1:15000)
Anti human Band 3, mouse monoclonal (BIII-136), Merck Sigma-Aldrich B9277 (1:15000)	HRP-conjugated goat anti-mouse IgGs, ENZO Life Sciences BML-SA204 (1:15000)
Anti human β-spectrin, mouse monoclonal (VD4), SCBT sc-53901 (1:2000)	HRP-conjugated goat anti-mouse IgGs, ENZO Life Sciences BML-SA204 (1:10000)
Anti human CD71, mouse monoclonal (H68.4), SCBT sc-51829 (1:200)	HRP-conjugated goat anti-mouse IgGs, Bio-Rad 170-6516 (1:4000)

Antibodies were diluted in a washing buffer [50 mM Tris/HCl pH 7.5, 0.2 M NaCl, 0.5 ml/l Tween 20, 1 g/l polyethylene glycol (PEG) 20000, 1 g/l BSA] that was also used for washing the membranes. The secondary horseradish-peroxidase-(HRP)-conjugated antibodies were used at the dilution indicated in Table 1. Membranes were developed with the chemiluminescence kit Prime Western Blotting Detection Reagent (GE Healthcare, United States) and the signal was acquired with a Molecular Imager ChemiDoc XRS + (Bio-Rad Laboratories, United States). Densitometry of the bands was performed using the software Scion Image (Scion Corporation, United States).

#### Antibodies Used

Mouse polyclonal anti human flotillin-2, H00002319-B02, Abnova, Taipei, Taiwan. Mouse monoclonal (BIII-136) anti human Band 3, B9277, Merck Sigma-Aldrich, Darmstadt, Germany. Mouse monoclonal (H68.4) anti human CD71, sc-51829; goat polyclonal anti human stomatin (M-14), sc-48308; mouse monoclonal anti human β-spectrin (VD4), sc-53901; HRP conjugated donkey anti-goat IgGs, sc-2020, Santa Cruz Biotechnology (SCBT), Dallas, TX, United States. HRP-conjugated goat anti-mouse IgGs, BML-SA204, ENZO Life Sciences, Lausanne, Switzerland. HRP-conjugated goat anti-mouse IgGs, 170-6516 Bio-Rad Laboratories, Milan, Italy. Antibodies were used at the dilutions given in Table 1.

### Membrane Raft Isolation as Detergent-Resistant Membrane

Membrane rafts were isolated from Retics and RBCs as detergent-resistant-membrane (DRM) according to our previously described protocol (Ciana et al., 2011). Due to the limited quantity of Retics available, a mini-preparation was performed. Briefly, the Retics obtained as described above by immunomagnetic sorting and a corresponding amount of RBCs from the same donor, were resuspended in HKM buffer (10 mM HEPES, 150 mM KCl, 4.5 mM NaCl, 1 mM MgCl_2_, pH 7.4, 300–315 mOsm/kg H_2_O) to a final volume of 30 μl. Five microliter were treated with SDS-PAGE sample buffer for Western blotting as described above. To 22.5 μl of Retic suspension in HKM, and 22.5 μl of RBC suspension in HKM (containing the equivalent of approximately 12.5 μl of packed RBCs), 37.5 μl of HKM, containing 1.67% (v/v) Triton X-100, were added at 4°C. The mixture was allowed to stand for 15 min on ice. Then, 62.5 μl of an 80% sucrose solution in 0.3 M K_2_CO_3_ were added to each sample for a total of 125 μl, and the mixtures transferred to the bottom of miniature ultracentrifuge tubes (Cod. 344090 Polyallomer Ultra-Clear, total volume 800 μl, 5 mm × 41 mm, Beckman Coulter, Milano, Italia). On top of the sample mixture, 350 μl of 30% sucrose in HKM were added, followed by a layer of 150 μl of 5% sucrose solution in HKM. The tubes were inserted into an adaptor to fit the rotor buckets used here (Delrin adapters, Cod. 356860 Beckman Coulter, Milano, Italia) and spun for 14 h at 225,000 × g_max_ at 4°C in a bench-top ultracentrifuge (Optima-Max, equipped with a swinging-arm MLS50 rotor, Beckman Coulter, Milan, Italy). At the end, six fractions of 100 μl each were collected from the top of the tube. The first five fractions were mixed each with 50 μl of 3X SDS-PAGE sample buffer, while the last fraction was mixed with 400 μl of “Diluted SDS-PAGE sample Buffer” (see above). All fractions were incubated at 60°C for 15 min, aliquoted and stored frozen.

## Results

### Filtration of Blood and Its Impact on Retic Yield

**FIGURE 1:**
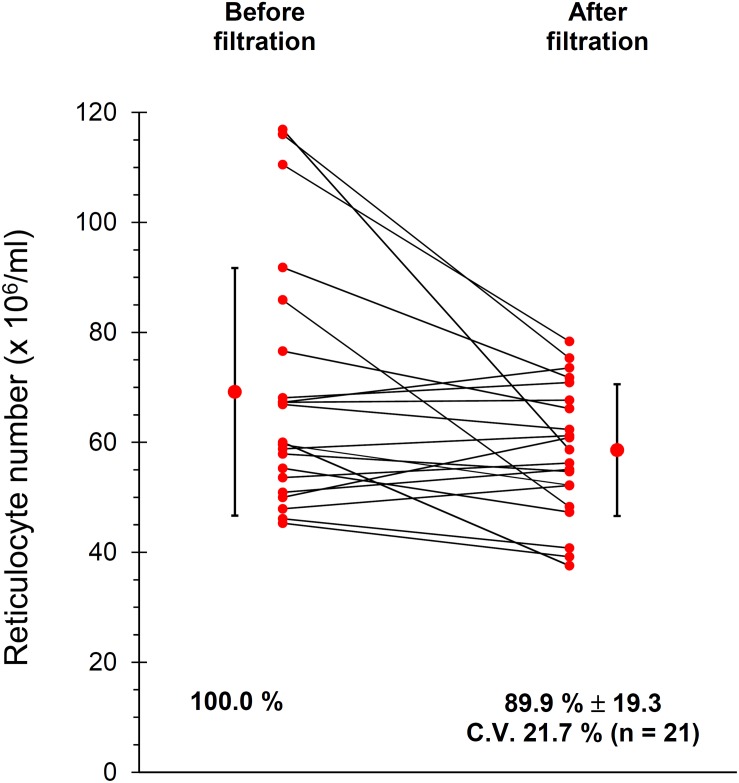
Recovery of Retics after filtration of whole blood through cellulose. Samples of cells as suspensions of approximately 20% Ht, before and after filtration through cellulose were analyzed in 21 independent experiments as described in Materials and Methods section. An average of approximately 10% of Retics was lost during filtration (*n* = 21, *p* = 0.014 at the paired Student’s *t*-test; CV, coefficient of variation).

Given the importance of working with purified RBC suspensions, free of contaminating leukocytes and platelets (Minetti et al., 2013) blood was leukodepleted by filtration through cellulose filters, as described in Materials and Methods section. The current protocol of filtration also includes a treatment of the filtered RBCs with diisopropylfluorophosphate (DFP) to inhibit the proteases from possible residual neutrophils. However, in preliminary experiments we observed that DFP impacts on the TfR of the Retic membrane, partially impairing the binding of anti CD71 antibodies and thus reducing the yield of the immuno-magnetic isolation of Retics (not shown). Therefore, in these experiments DFP treatment was omitted. Although the original article (Beutler et al., 1976) excluded loss of Retics during filtration through cellulose, we decided to confirm this observation in our setup. The results shown in Figure 1 revealed that only approximately 10% of Retics remained trapped in the filter.

### Quantification of Flotillin-2 and Stomatin Relative to Band 3 in Retics and RBCs

**FIGURE 2:**
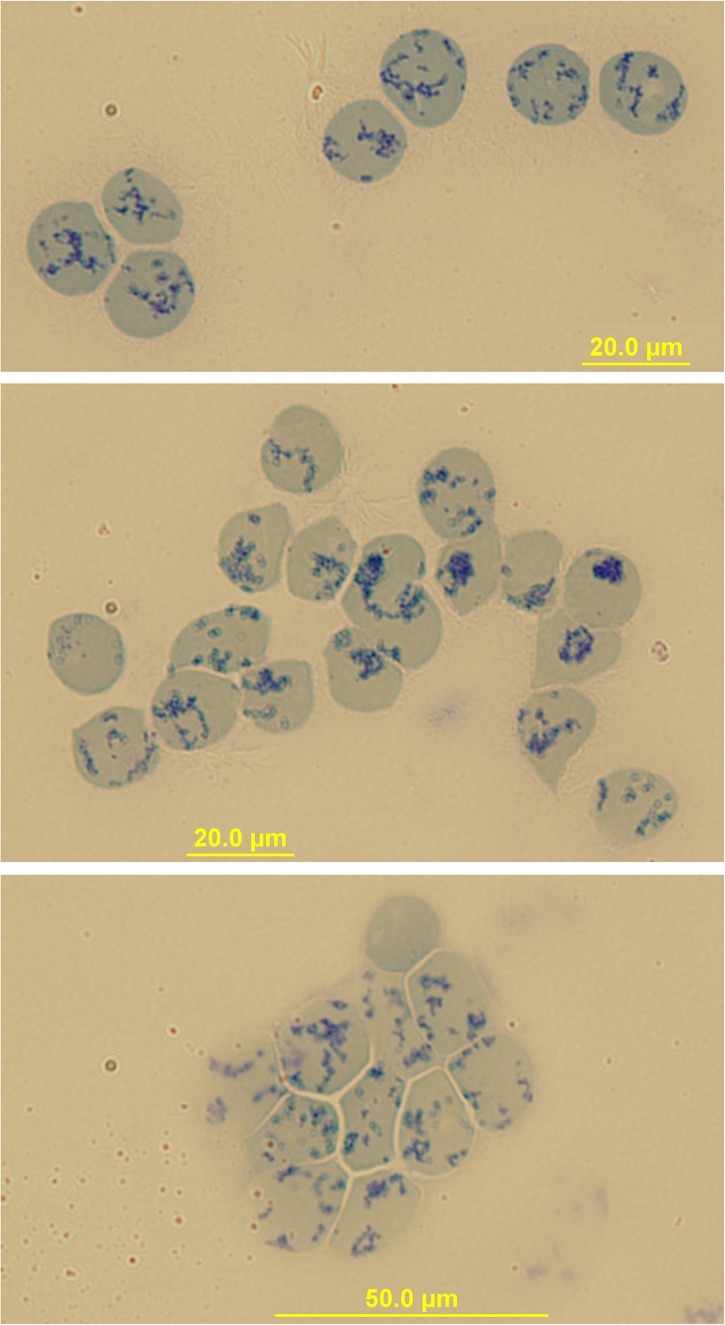
Immunomagnetical isolation of peripheral blood Retics. Images of Retics after isolation with “CD71-microbeads” and staining with New Methylene Blue. Sometimes clumps of Retics are visible (bottom image) probably due to a crosslinking effect of the microbeads.

**FIGURE 3:**
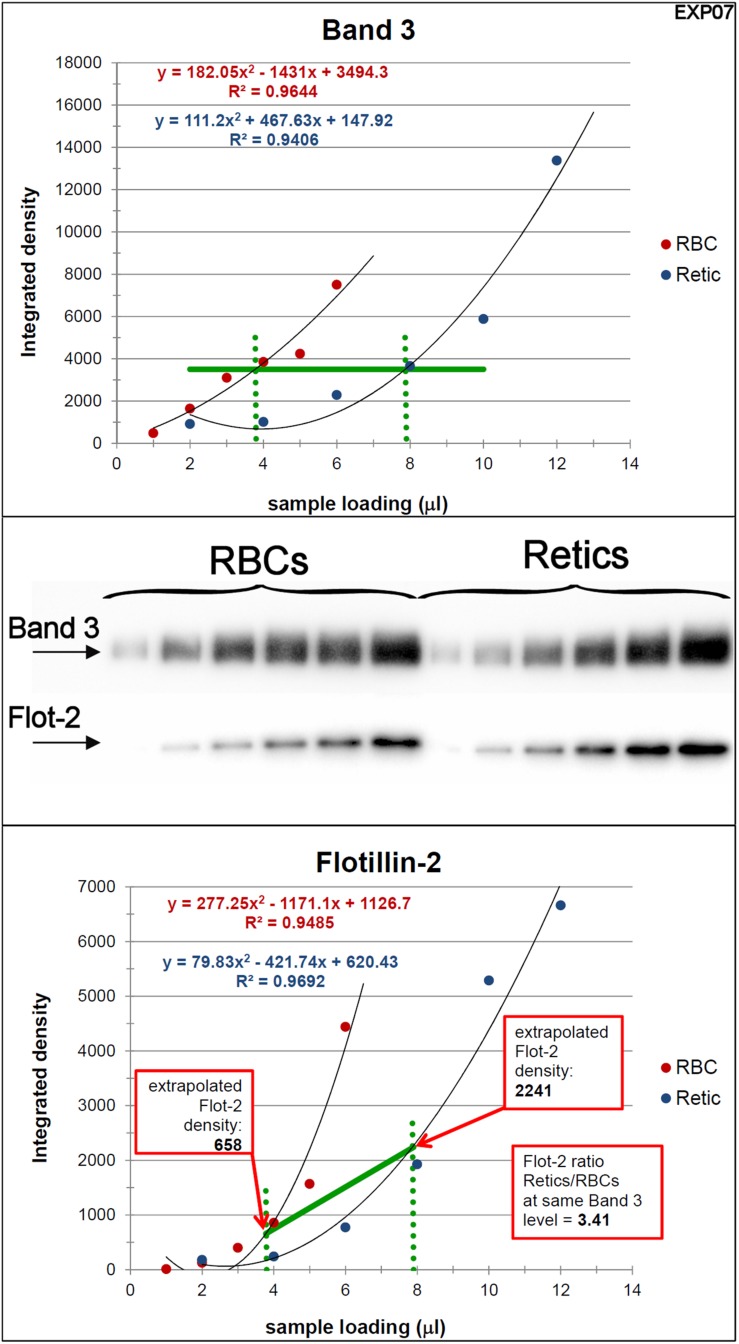
Quantification of the relative amounts of flotillin-2 with respect to Band 3 in Retics and RBCs. The integrated densities of Band 3 obtained by densitometry of the Western blotting membrane shown in the figure (the two series of RBC and Retic samples were loaded in the same gel) were fitted with a polynomial curve (2nd order), one for the RBC and one for the Retic series (Band 3 graph). The two curves were intersected with a horizontal line at a Y value taken approximately mid-way on the polynomial curves (horizontal green line in the Band 3 graph). From the intercepts between the polynomials and the horizontal green line, vertical lines (dotted green lines) were projected on the X axis. The intercepts on the X axis were taken as the loading values (in μl) that would give the same intensity (amount) of Band 3 for RBCs and Retics. These values were used to extrapolate, from the graph where flotillin-2 integrated densities were similarly interpolated by a parabolic function (flotillin-2 graph), the flotillin-2 densities expected for an equal amount of Band 3 in RBCs and Retics. The ratio between the flotillin-2 density extrapolated on the Retic curve and that extrapolated on the RBC curve gave the ratio of flotillin-2 between Retics and RBCs at the same level of Band 3. This procedure was repeated in 11 independent experiments (see detailed data in Supplementary Material).

**FIGURE 4:**
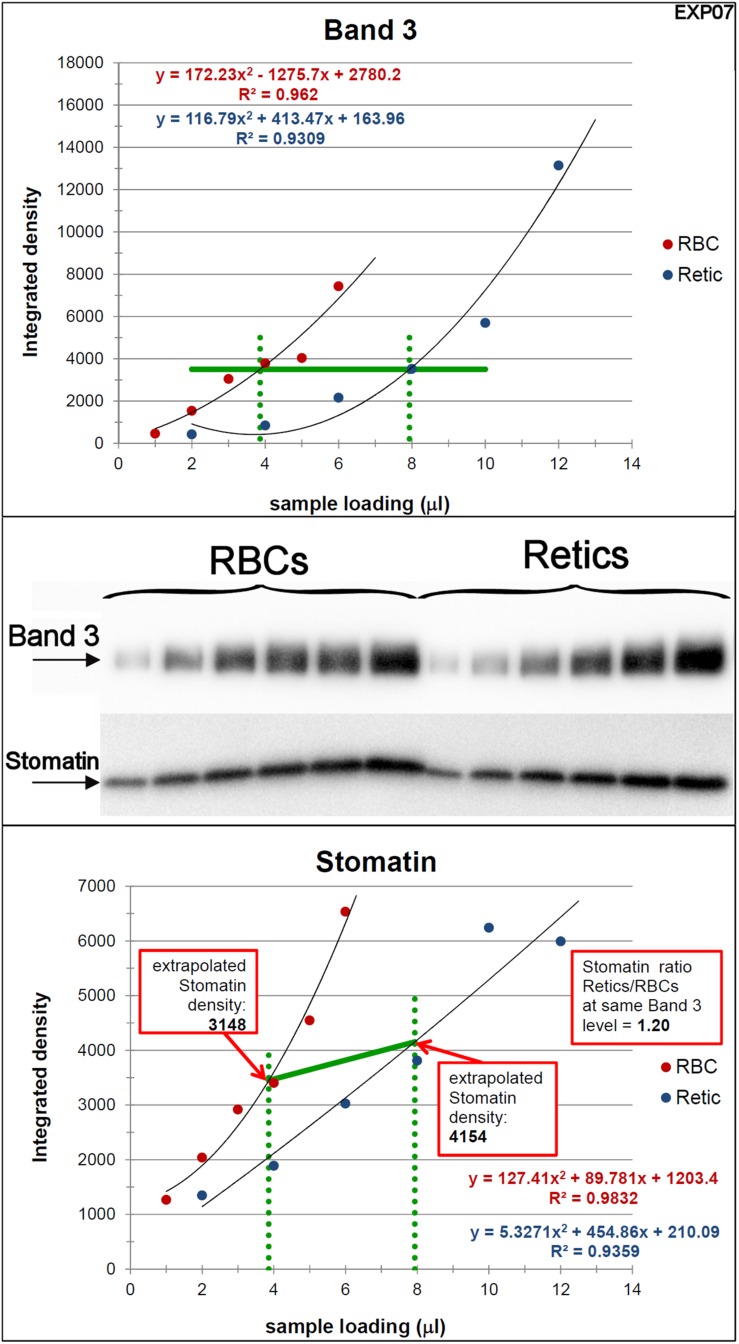
Quantification of the relative amounts of stomatin with respect to Band 3 in Retics and RBCs. The same procedure adopted for flotillin-2 was performed to quantify the levels of stomatin in Retics and RBCs from the same donor. See legend to Figure 3 for details.

Peripheral blood R2 Retics were isolate in pure form with the immunomagnetic method described in Materials and Methods section and processed as such while still attached to the “CD71 Microbeads” (Figure 2). Membrane rafts are known to be involved in the sorting of proteins that occurs during the maturation of R1 Retics, which is reflected in the release of exosomes enriched in proteins and lipids that are typical constituents of rafts (Skotland et al., 2017). Flotillins and stomatin are highly enriched in membrane rafts (de Gassart et al., 2003; Ciana et al., 2014). We have previously observed that especially flotillin-2 is lost in a disproportionate manner with respect to the loss of membrane surface area that takes place during the physiological aging of RBCs, suggesting an involvement of membrane rafts also in this physiological age-related remodeling of the RBC membrane (Ciana et al., 2017a). We therefore wondered whether membrane rafts are also in some way selected for removal from the membrane of R2 Retics. An analysis of the relative abundance of flotillin-2 and stomatin with respect to Band 3 in pure Retics and RBCs from the same donor was therefore conducted. This quantification was approached by Western blotting. Quantification of a given protein by densitometric analysis of a Western blotting membrane cannot be considered reliable in the absence of a calibration standard with known amounts of the protein of interest. To circumvent this problem and reduce the error, quantification was performed by loading in the same electrophoretic gel increasing amounts of each of the two samples to be compared (RBCs and Retics). Two identical gels were loaded in this way and electro-transferred to two PVDF membranes. One membrane was probed for Band 3 and the other for flotillin-2 or stomatin. Preliminary tests were conducted to ensure a proper sample loading so that the detected signal never reached saturation. Digital images from the Western blotting were subjected to densitometric analysis to measure the integrated density of each band. The normalization of flotillin-2 and stomatin levels over Band 3 levels was conducted as shown in Figures 3, 4, respectively, and described in the legend to Figure 3. For flotillin-2, the results obtained in 11 independent experiments were averaged and are reported in Table 2. It can be observed that flotillin-2 levers are on average 2.5 times higher in Retics than in RBCs when normalized over Band 3 levels.

**TABLE 2:** Western blotting quantification of the ratio between flotillin-2 and stomatin in Retics and in RBCs normalized over identical amounts of Band 3, as determined by the procedure described in the text.

	**Retics/RBCs (*n* = 11)**	**Retics/RBCs (*n* = 6)**	**UNPAIRED *t***-**test**		
	**FLOT-2**	**STOM**	**FLOT-2 vs**. **STOM**	**FLOT-2 vs**. **1**	**STOM vs**. **1**
MEAN	2.55	1.49	*p* < 0.05	*p* < 0.0001	*p* > 0.05
SD	0.93	0.63			
CV%	36.5	42.6			

For stomatin, the results collected in six independent experiments are reported in Table 2. Stomatin levels are on average 1.5 times higher in Retics than in RBCs when normalized over Band 3. After statistical analysis, it emerged that, whereas flotillin-2 levels are significantly higher in Retics than in RBCs relative to the same amounts of Band 3 from each cell type, those of stomatin are not significantly different between RBCs and Retics (not different from a ratio of 1:1). Moreover, flotillin-2 and stomatin values are significantly different from each other (for details see also Supplementary Material, Sheet “SUMMARY”).

### Estimation of the Relative Levels of Band 3 per Cell in Retics and RBCs

We initially assumed that the number of copies of Band 3 per cell remains constant also in the maturation of R2 Retics to RBCs, as it does in the maturation of R1 Retics (exosomes don’t contain Band 3). We reasoned that even if the assumption was incorrect, and Band 3 was to some extent lost with the membrane of maturing R2 Retics, a decrease in flotillin-2 or stomatin relative to Band 3 from Retics to RBCs would be at most underestimated. We wanted, however, to gain more insight on this issue and performed a new Western blotting quantification by loading the same number of RBCs and Retics and evaluating the levels of Band 3 and β-spectrin, as representative of the most abundant membrane-skeletal proteins. Results obtained in two independent experiments showed that Band 3 levels in Retics are 2.48 times higher than in the same number of RBCs (individual measurements deviated from the mean by 0.7%). When β-spectrin was analyzed by the same method, it turned out that Retics have 2.19 times more than RBCs (individual measurements deviated from the mean by 21%) (see Supplementary Material, Sheet “EXP09-10-B3-Sp”).

### Partition of TfR in Membrane Rafts in Retics and RBCs

**FIGURE 5:**
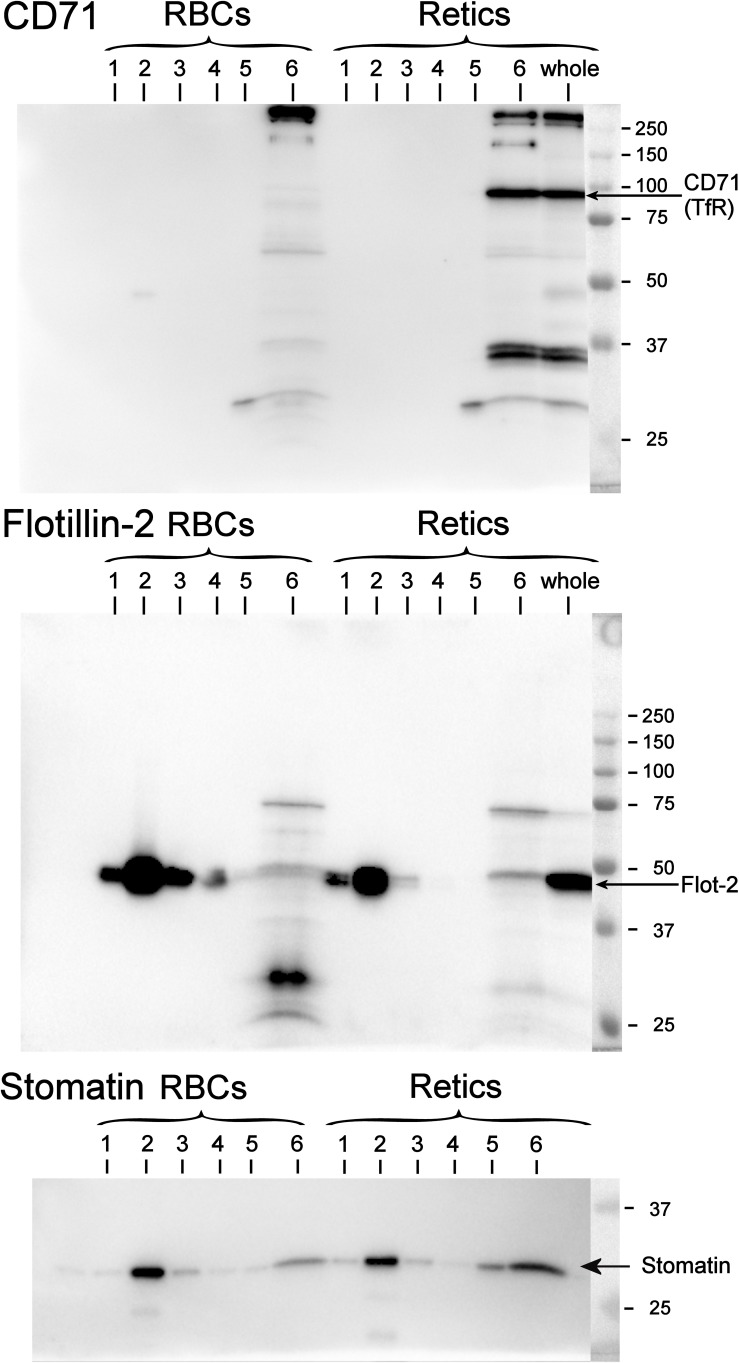
Partitioning of TfR, flotillin-2 and stomatin between the raft and non-raft phase in RBCs and Retics. Six fractions of a sucrose gradient from a mini-preparation of membrane rafts as DRM from Retics and RBCs were probed by Western blotting for CD71, flotillin-2 and stomatin. Membrane rafts partition in fraction 2 from the top of the gradient, as shown by the presence of flotillin-2 and stomatin in this fraction for both Retic and RBC samples. In the Retic sample the TfR is only detected in the non-raft fraction at the bottom of the gradient (fraction 6). “Whole” is a sample of whole Retics. See text for details.

It is known that, although TfR and membrane raft components are together present in exosomes, the TfR itself does not co-localize with other membrane raft proteins in the plasma membrane. Therefore, diverse endocytic pathways that lead to the formation of a late endosomal compartment (then becoming a MVB from which the ILV/exosomes originate) must exist, carrying, on the one hand the TfR and, on the other, raft components to the MVB (see Minetti et al., 2018 for a discussion of this issue). To the best of our knowledge, the partitioning of TfR between the membrane and rafts phase in human R2 Retics has not been investigated before. It could be that due to the profound rearrangements in the architecture of the membrane that occur in the maturation of R1 Retics, some constraints are relieved that excluded the TfR from the rafts, allowing it to partition into these membrane domains in more mature Retics. We addressed this issue by isolating membrane rafts as DRM according to the correct procedure in the absence of proteolysis and with the use of carbonate (Ciana et al., 2011). A miniaturized protocol for DRM isolation was adopted due to the scarce quantity of Retics available. In Figure 5 the results of the Western blotting for flotillin-2, stomatin and CD71 (TfR) of DRM fractions from RBCs and Retics are shown. It can be concluded that, unlike flotillin-2 and stomatin, which partition almost exclusively in the DRM region in both Retics and RBCs, TfR is totally absent from the DRM in Retics (in mature RBCs no TfR could be expected to be detected).

### Lipidomics of Retics and RBCs

Proceeding from the evidence of a selective decrease in flotillin-2 and, although to a lesser extent, stomatin relative to Band 3 in the maturation from Retics to RBCs, and being flotillins and stomatin established membrane raft markers (Salzer and Prohaska, 2001), we hypothesized that the analysis of membrane lipids in Retics and RBCs could reveal a similar decrease of those lipids that are described as being enriched in membrane rafts, namely sphingolipids and cholesterol, and the ratio (sphingolipids + Chol)/phospholipids would decrease accordingly. Lipids from pure Retics and RBCs were therefore analyzed in five independent experiments, the results are shown in Table 3. It can be observed that, contrary to what hypothesized, sphingolipids and Chol actually increased relative to total lipids, in a statistically significant way, from Retics to RBCs. Conversely, PC and PS decreased. The ratio (SM + Chol)/phospholipids changed from 1.69 ± 0.10 in Retics to 2.00 ± 0.09 in RBCs (*n* = 5; *p* = 0.003).

**TABLE 3:** Quantification of lipids in the membrane of Retics and RBCs in five independent experiments with five different donors.

		**RETIC**	**RBC**	***p-*value**
−	PC	24.0 ± 1.3	20.5 ± 1.1	0.007*
	LPC	1.2 ± 0.2	1.2 ± 0.2	0.910
+	SM	17.2 ± 0.8	19.8 ± 1.0	0.015*
	PE	7.1 ± 2.1	7.5 ± 0.9	0.539
−	PS	4.2 ± 0.5	3.5 ± 0.5	0.010*
	PI	0.7 ± 0.2	0.7 ± 0.2	0.559
+	Chol	45.6 ± 1.2	46.8 ± 0.8	0.041*

**Values are mean ± standard deviation and are expressed as mol% over the total lipids analyzed in each sample of Retics or RBCs. The symbols in the left column indicate whether the quantity of a given lipid increases (minus) or decreases (plus) from Retic to RBC. To the right, the p values obtained from the statistical elaboration of the data with the Student’s t-test are shown. An asterisk indicates where p < 0.05.**

Tables 4–6 report the relative abundance of the subclasses for those lipids that changed most in the maturation of Retics: PC, SM, and PS. It is worth mentioning, in particular, that the subclasses of SM are represented differently in Retics and RBCs, whereby SM with saturated acyl chains are more abundant in RBCs than in Retics. In Retics, unsaturated acyl chains prevail in SM (Table 4).

**TABLE 4:** SM subclasses in Retics and RBCs subjected to lipidomics analysis.

		**RETIC**	**RBC**	***p*-value**
+	SM 14:0	1.9 ± 0.2	2.0 ± 0.3	0.014*

+	SM 15:0	1.3 ± 0.1	1.4 ± 0.1	0.002*

+	SM 16:0	22.6 ± 1.8	24.4 ± 2.5	0.020*

+	SM 16:1	1.8 ± 0.3	2.0 ± 0.3	0.002*

+	SM 17:0	0.5 ± 0.1	0.7 ± 0.1	0.003*

+	SM 18:0	2.1 ± 0.2	3.7 ± 0.3	0.000*

+	SM 18:1	0.8 ± 0.1	1.3 ± 0.1	0.000*

+	SM 20:0	1.1 ± 0.2	1.2 ± 0.2	0.030*

−	SM 22:0	8.6 ± 0.9	8.1 ± 0.9	0.074

−	SM 22:1	3.1 ± 0.5	2.7 ± 0.4	0.004*

−	SM 23:0	2.0 ± 0.3	2.0 ± 0.3	0.318

−	SM 24:0	18.1 ± 1.5	17.8 ± 1.7	0.636

−	SM 24:1	28.1 ± 2.5	26.0 ± 2.1	0.005*

−	SM 24:2	7.0 ± 0.6	5.5 ± 0.4	0.001*

+	SM 26:0	1.0 ± 0.1	1.1 ± 0.1	0.035*

+	SATURATED	59.1 ± 2.2	62.5 ± 2.1	0.003*

−	MONO	33.8 ± 2.5	32.1 ± 2.1	0.014*

−	PUFA	7.0 ± 0.6	5.5 ± 0.4	0.001*

−	UNSATURATED	40.9 ± 2.2	37.5 ± 2.1	0.003*

				

**Values of each lipid subclass are expressed as mol% over the total SM. Numbers in the name of each SM species indicate the length and the number of unsaturations in the acyl chain N-amidated to the sphingosine moiety. The four rows at the bottom group, respectively, all the subclasses of SM with saturated acyl chains (SATURATED), those with a mono-unsaturated acyl chain (MONO), those with an acyl chain with more than one double bond (PUFA) and the total of unsaturated species (MONO + PUFA) (UNSATURATED). The symbols in the left column indicate whether the quantity of a given lipid increases (minus) or decreases (plus) from Retic to RBC. To the right, the p values obtained from the statistical elaboration of the data with the Student’s t-test are shown. An asterisk indicates where p < 0.05.**

For PC, which in total decreased from Retics to RBCs, the opposite is true, with saturated and mono-unsaturated species contributing more to the decrease and poly-unsaturated species prevailing in RBCs over total PC forms (Table 5).

**TABLE 5:** PC subclasses in Retics and RBCs subjected to lipidomics analysis.

		**RETIC**	**RBC**	***p*-value**
−	PC 32:0	13.6 ± 3.8	6.6 ± 1.2	0.005*

+	PC 32:1	1.4 ± 0.5	1.5 ± 1.0	0.698

−	PC 33:0	1.4 ± 0.2	1.0 ± 0.1	0.012*

−	PC 34:0	2.8 ± 0.2	2.4 ± 0.3	0.030*

−	PC 34:1	33.6 ± 3.4	25.7 ± 2.2	0.001*

+	PC 34:2	13.1 ± 1.9	23.3 ± 1.3	0.000*

−	PC 35:0	1.4 ± 0.3	1.0 ± 0.0	0.069

−	PC 35:1	1.1 ± 0.2	0.9 ± 0.1	0.015*

+	PC 35:2	0.5 ± 0.1	0.5 ± 0.0	0.242

−	PC 35:3	0.5 ± 0.2	0.3 ± 0.0	0.093

−	PC 35:4	0.5 ± 0.2	0.3 ± 0.0	0.066

+	PC 36:1	3.9 ± 0.6	6.1 ± 1.0	0.001*

+	PC 36:2	6.3 ± 1.1	10.9 ± 1.8	0.000*

+	PC 36:3	4.3 ± 0.7	5.8 ± 0.4	0.001*

−	PC 36:4	6.4 ± 0.9	5.8 ± 1.0	0.375

−	PC 37:3	4.9 ± 1.7	3.1 ± 0.2	0.069

−	PC 37:5	0.6 ± 0.3	0.3 ± 0.0	0.079

+	PC 38:3	0.4 ± 0.1	0.8 ± 0.2	0.019*

−	PC 38:4	2.4 ± 0.5	2.4 ± 0.4	0.914

+	PC 38:6	1.0 ± 0.1	1.2 ± 0.6	0.275

−	SATURATED	19.1 ± 3.6	11.0 ± 1.3	0.002*

−	MONO	40.1 ± 3.2	34.3 ± 1.6	0.004*

+	PUFA	40.8 ± 6.3	54.8 ± 2.4	0.002*

				

**Values of each lipid subclass are expressed as mol% over the total PC. Numbers in the name of each PC species indicate the length and the number of unsaturations in the acyl chains esterified to the glycerol backbone. Please refer to the legend of Table 4 for additional details.**

Finally, although a statistically significant overall decrease of PS over total lipids was observed in the maturation to RBCs, the minor differences that were observed in the relative abundance of the two major species of PS were not statistically significant (Table 6).

**TABLE 6:** PS subclasses in Retics and RBCs subjected to lipidomics analysis.

		**RETIC**	**RBC**	***p*-value**
+	PS 38:3	2.0 ± 0.5	2.9 ± 2.2	0.453
−	PS 38:4	75.4 ± 11.5	74.0 ± 10.0	0.793
+	PS 40:6	22.6 ± 11.7	23.1 ± 9.2	0.906

**Values of each lipid subclass are expressed as mol% over the total PS. Numbers in the name of each PS species indicate the length and the number of unsaturations in the acyl chains esterified to the glycerol backbone. Please refer to the legend of Table 4 for additional details.**

## Discussion

The series of maturational processes that RBCs and Retics undergo *in vivo* cannot be studied by analyzing the material that is lost in the circulation, because this is cleared before it could be recovered from plasma (Ciana et al., 2017a). Therefore, the only hints could be obtained from the comparison of the membrane at the beginning and end of the process, be this the maturation of R2 Retics to RBCs or the aging of the circulating RBCs, with all the inaccuracy and lack of sensitivity associated with the quantification of differences between large numbers. Yet, by applying this method differences could be detected, and we have recently proposed that a continuous process of maturation that involves the progressive loss of membrane raft components characterizes the life of RBCs from the stage of young cells in the circulation to clearance (Minetti et al., 2018). The selective loss of membrane rafts and selected membrane proteins (i.a. the TfR) with exosomes and the complete retention of the membrane-skeleton and the main integral membrane proteins (Band 3, glycophorins) was already amply demonstrated in the maturation of R1 Retics (de Gassart et al., 2003). The available results of lipid analysis are less consistent in clearly showing the paradigmatic enrichment of raft lipids in the released exosomes, in particular from Retics. This could be also due to the paucity of studies that have addressed this issue. In one study, only a minor enrichment in SM in the exosomes released *in vitro* by maturing guinea pig Retics was reported (Vidal et al., 1989). A more recent high-resolution lipidomics study conducted on rat “stress” Retics revealed a more complex scenario, whereby the lipid composition of exosomes varied at various stages of maturation of the Retics (Carayon et al., 2011). Therefore it is still unclear whether the lipid composition of exosomes really reflects, as the protein components apparently does, the enrichment in membrane rafts in this type of microvesicles, at least for the Retic (Skotland et al., 2017). Independently from what happens to the lipid component in the maturation of R1 Retics, we have observed here a profound rearrangement of membrane lipids with the significant increase in SM and Chol and the decrease in PC and PS in the maturation of circulating R2 Retics, that translated into a highly significant increase in the ratio (SM + Chol)/phospholipids. We initially hypothesized that this ratio should decrease in the maturation of R2 Retics. In fact, our recent study of young and old RBCs revealed that a disproportionate loss of flotillin-2 and stomatin takes place during the aging *in vivo* of RBCs (Ciana et al., 2017a). We inferred from this that all membrane raft components could be selectively lost, pointing to a possible involvement of membrane rafts in the age-related remodeling of the RBC membrane in the circulation.

By examining here the disproportionate loss of flotillin-2 and, to a lesser extent, of stomatin relative to Band-3 from Retics to RBCs, we initially recognized the hypothesized continuum of selective decrease in membrane raft components. When challenged with the analysis of lipids, however, this tenet could no longer be sustained because of the actual increase, instead of the expected decrease in the relative abundance of membrane raft lipids in the mature RBC with respect to the R2 Retic, as if RBCs were enriched in membrane rafts with respect to Retics. The two results are clearly conflicting and their interpretation is challenging (see below for additional comments).

The literature abounds with report of protein quantification carried out by Western blotting. In the light of results obtained here, we caution against the fallacious assumption that is at the basis of such quantifications, i.e., that the chemiluminescence signal obtained by Western blotting is linearly proportional to the quantity of protein. To at least partially solve this problem, we have here adopted a different approach in which signal response curves were fitted with parabolic functions. In our particular setup, the best fitting curve was used on the Band 3 blots to determine the sample volume where identical quantities of Band 3 were present for RBCs and Retics. These values were then used to extrapolate the intensity signal of flotillin-2 (or stomatin) for Retics and RBCs on parabolic curves that fitted the flotillin-2 (or stomatin) signal obtained from the series of loaded samples. The ratio between these two signal values was then calculated and trusted as the real quantitative relation of the protein of interest between Retics and RBCs. However, it must be remarked that these two values of intensity signal lie on a curve which is in itself non-linear, and therefore their ratio is an overestimation of the real value. In other words, with this method the differences between samples are amplified to an extent proportional to the deviation from linearity of the “dose-response” curve. A value of 2.5 times more flotillin-2 in Retics than RBCs relative to Band 3 (or a value of 1.5 for stomatin) is therefore most likely over-estimated because of this distortion.

Quantification of Band 3 in experiments where the same number of cells (Retics and RBCs) were loaded (see Supplementary Material, Sheet “EXP09-10-B3-Sp”) revealed a decrease that, according to the Western blotting results, also appeared to be disproportionate to the difference in surface area between Retics and RBCs. Band 3 levels appear to be 2.5 higher in Retics than in RBCs from the same donor. As it is unlikely that R2 Retics lose more than half of their membrane in maturing to RBCs, the Band 3 difference is probably overestimated, due to the non-linearity of Western blotting quantification. Yet, as the differences in flotillin-2 and stomatin between Retics and RBCs were evaluated relative to Band 3, they must reflect a real selective loss of the two proteins (higher for flotillin-2, lower for stomatin), disproportionate with respect to the loss of membrane surface area and of Band 3.

Another comment on Band 3 quantification, is that a difference of approximately 30% in the levels of Band 3 between two donors when the same numbers of cells were loaded in the gels was observed, both for RBCs and Retics. The mean cell volume (MCV) difference of RBCs from the two donors was in the same direction as the Band 3 difference (higher MCV for the donor with more Band 3), but it amounted to no more than 1%. As it is unlikely that copy numbers of Band 3 molecules per RBC in the general population differ by as much as 30%, this value must have been again amplified because of the non-linear bias intrinsic in the Western blotting quantification.

Concerning the observed decrease relative to Band 3, in the raft marker protein flotillin-2 (and, to a lesser extent in stomatin), and the conflicting result of an apparent relative increase in raft lipids from Retics to RBCs, additional comments are in order. Considering the never settled debate as to whether DRM are a valid representation of membrane rafts, it could be questioned whether flotillins are indeed a component of membrane rafts, because, from our results, they seem to be lost independently from the typical raft lipids. Although the literature on the localization of flotillins in cell membranes provides evidence of a selective partitioning of these proteins in the liquid ordered phase, this notion is largely derived from studies conducted on DRM that assume the identity of DRM and membrane rafts with all the limitations associated with this questioned protocol. Flotillin-1 defines a form of clathrin-independent endocytosis, different form the fluid phase endocytosis dependent on caveolae (Glebov et al., 2006). Consistent with this, it is found localized in different rafts from those where caveolins are found. Whether flotillins are also capable of driving these forms of endocytosis, and if they exist in RBCs is unknown. It was earlier hypothesized that membrane rafts are heterogeneous entities (Pike, 2004). It could be therefore that different raft families exist, some in which flotillins and stomatin are absent, and some with variable and different amounts of each of these two proteins. This different distribution could be driven by a different lipid composition of the rafts, perhaps also in terms of different phospholipid subclasses. Whereas, all the raft classes could be isolated as DRM with the non-ionic detergent; only one or more subclasses are indeed lost with the maturation of Retics, taking with them a significant portion of flotillin-2 and stomatin. The larger difference in flotillin-2 content than in stomatin content between Retics and RBCs that we have documented here seems to support this view. It is always possible, on the other hand, that flotillins decrease through mechanisms independent from the loss of membrane extension (e.g., proteolysis, aggregation). That flotillins behave differently from other membrane raft proteins is suggested by our own results on the above-mentioned disproportionate decrease in flotillin-2 with RBC aging. In the same study, the decrease in stomatin from young to old RBCs was much less pronounced than that of flotillin-2, and almost entirely justifiable by the loss of membrane surface area from young to old RBCs (Ciana et al., 2017a). Future investigations into this aspect will require the analysis of a wider range of raft proteins and lipids in Retics and RBCs. Also, lipidomics analysis of RBCs of different age is in order to ascertain whether there is a remodeling of the lipid components with the aging of RBCs.

Results of the lipidomics analysis of the different lipid subclasses revealed significant differences between Retics and RBCs. In front of an increase of total SM and also of all the SM subclasses with respect to total membrane lipids (about 2.7% of total lipids) from Retics to RBCs, the relative abundance of the various subclasses changed significantly in this maturation process. The species with a shorter and saturated N–amidated acyl chain increased more than the unsaturated species so that the ratio saturated/unsaturated became higher in RBCs. Scarce are literature data on the different lipid subclasses in membrane rafts. In one study, DRMs from human RBC ghosts were shown to be enriched in the saturated species of SM and depleted in the unsaturated ones (Koumanov et al., 2005). Our results would therefore corroborate the view of the retention of membrane rafts in the maturation of Retics to RBCs. Less clear are the changes in PC content. Here, an overall decrease of PC of about 3.5% from Retics to RBCs would still be compatible with the retention of membrane rafts in the transition Retic to RBC, because the percentage of PC over total lipids is lower in rafts than in the parent cell membrane. Therefore a preferential loss of non-raft material from Retics would be compatible with the overall decrease in PC observed here. Looking at the reorganization of PC subclasses in our data set, more subtle and interesting differences emerge. The saturated and mono-unsaturated PC species decrease more in RBCs than the polyunsaturated species. Assuming that the PC content in rafts is represented more by saturated and monounsaturated species, as could be inferred from data obtained in other cell types (Ogiso et al., 2015), our data shows an enrichment in RBCs of the polyunsaturated PC classes which go against the above hypothesized retention of rafts in the maturation of Retics to RBCs. However, the composition of rafts varies widely in different cell types, the distribution of PC subclasses in raft and non-raft human RBC samples was not documented in the mentioned article (Koumanov et al., 2005) and we could not find a source for this information in the literature. Therefore, this interpretation is not final. It could be also conceived that with the preferential decrease in saturated PC species, the unsaturated ones would contribute to an overall fluidification of the membrane in mature RBCs.

**FIGURE 6:**
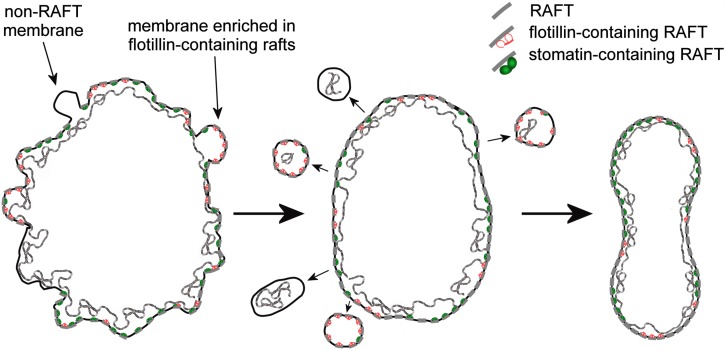
Graphical conceptualization of the process of membrane raft enrichment in the maturation of R2 Retics. From a large and irregularly shaped R2 Retic (left) selected portions of non-raft membrane, or membrane selectively enriched in flotillin-2-containing rafts, together with some membrane skeleton are lost or, more likely, pinched off in the splenic milieu by the action of macrophages. The now resized lipid bilayer (middle) completes the anchoring to the membrane skeleton also thanks to the contribution of membrane raft interaction with the spectrin network, resulting in a mature and stable discocytic shape (right). The anchoring complexes based on band 3-ankyrin and the actin-based junctional complexes have been omitted for clarity. Membrane rafts are shown as short segments crossing the lipid bilayer. The various components are not drawn in scale.

From the reassortment of lipids between R2 Retics and RBCs it could be concluded that membrane rafts are relatively enriched in RBCs. It remains to be established whether the same enrichment occurs during the aging of RBCs in the circulation, and future work will fill this gap. In our previous research on membrane rafts, isolated as DRM from human RBC ghosts (Ciana et al., 2005; Crepaldi Domingues et al., 2009) or whole RBCs (Domingues et al., 2010; Ciana et al., 2011, 2013) we have amply demonstrated that for the extraction of DRM, an increase in pH and ionic strength of the medium containing the non-ionic detergent (Triton X-100) must be also provided. This requirement was interpreted as if electrostatic interactions existed, determining the association between membrane rafts and the membrane-skeleton (Ciana et al., 2014). This pointed to the existence of proteolipid interactions between the bilayer and the spectrin network that contribute, beside the conventional protein-mediated anchorages, to the stabilization of the membrane. Such high-salt-sensitive association between raft proteins and the membrane skeleton was recently confirmed in a proteomic analysis of triton-insoluble membrane skeletons from human RBCs (Basu et al., 2015). Maturing Retics are highly unstable, because the lipid bilayer is not yet completely anchored to the skeleton, and an excess membrane must be eliminated. The selective retention of membrane rafts in mature RBCs may find an explanation in the role of the rafts as additional anchoring sites that associate the bilayer with the skeleton. After the rafts, or at least those raft subclasses that have this ability, become associated with the spectrin network, the excess of plasma membrane that is not linked with the skeleton could be lost. Because this extra membrane is relatively depleted in rafts, the lipid composition of the bilayer in the mature RBC will reflect this selective rearrangement with the increase in the relative content of SM and Chol, as we have shown here. A graphical conceptualization of the model of Retic maturation proposed here is shown in Figure 6.

Other aspects have emerged from this study that are connected with the relative quantification of Band 3 and spectrin in Retics vs. RBCs. With all the limitations associated with the non-linearity of Western blotting quantification, results showed nonetheless that Band 3 and spectrin seem to be partially lost with the membrane of maturing R2 Retics. With this approach we could not assess whether β-spectrin is lost together with portions of the membrane skeleton, or as isolated heterodimers with α-spectrin or tetramers. From results on the maturation of R1 Retics, where, despite the significant loss of plasma membrane, all membrane-skeletal proteins are retained together with Band 3, it was expected that also for the maturation of R2 retics an efficient method was selected to salvage important and precious macromolecules that could all contribute to defining the final architecture of the mature RBCs. This loss of membrane skeleton was therefore somewhat unexpected, and if confirmed, it will thicken the mystery surrounding the mechanism(s) of terminal Retic maturation. In fact, it is still unclear if, and to what extent Retics can complete maturation *in vitro* (Gronowicz et al., 1984; Koury et al., 2005). In our opinion it is unlikely that membrane loss is a process that R2 Retics can perform spontaneously. In support to this concept there are two major observations: in splenectomized subjects, maturation of Retics is delayed and altered and RBCs display morphological and biochemical alterations (Holroyde and Gardner, 1970; Shattil and Cooper, 1972; Crosby, 1977; De Haan et al., 1988) *in vitro* cultured RBCs fail to achieve complete maturation to biconcave discocytes (Giarratana et al., 2005; Shah et al., 2014). In an early report it was described that Retics contain a high molecular weight complex, composed of spectrin and other not better characterized membrane proteins, that is lost with maturation only in normal subjects, whereas it is still present in circulating RBCs in splenectomized patients (Lux and John, 1977). This membrane remodeling was interpreted as the result of a “culling” action of the spleen. This organ would be continuously processing the cells and be responsible for generating those changes that R2 Retics and RBCs sustain throughout their circulatory life, simply because these cell types are no longer able to remodel their structure spontaneously. Shedding light on basic mechanisms of membrane remodeling, on the role of lipids in erythropoiesis, and on the interplay between RBCs and other components of the vascular system may also contribute to improve the conditions for the cultivation and full maturation *in vitro* of RBCs for transfusion purposes.

## Data Availability Statement

The datasets generated for this study are available on request to the corresponding author.

## Ethics Statement

The studies involving human participants were reviewed and approved on 2017/04/10, by the local ethics committee: Comitato Etico Area Pavia, IRCCS Policlinico San Matteo, Pavia, Italy. The patients/participants provided their written informed consent to participate in this study.

## Author Contributions

GM, AC, and CA contributed to the conception and design of the work. GM, CA, AC, CB, and ID contributed to the experimental work. CB and ID contributed to the lipid analyses. GM, AC, CA, CB, ID, SB, and CP contributed to the data analysis and interpretation. GM and AC contributed to the writing of the manuscript. CB, ID, SB, and CP contributed to the editing of the manuscript. All authors edited and revised the manuscript, and gave final approval for publication.

## Conflict of Interest

The authors declare that the research was conducted in the absence of any commercial or financial relationships that could be construed as a potential conflict of interest.

## Abbreviations

Chol: cholesterol
DRM: detergent-resistant-membrane
ILV: intraluminal vesicle
LPC: lysophosphatidylcholine
MVB: multivesicular body
PC: phosphatidylcholine
PI: phosphatidylinositol
PS: phosphatidylserine
SM: sphingomyeline.
